# Supplementary material for: Fabrication of Superhydrophobic/Superoleophilic Bamboo Cellulose Foam for Oil/Water Separation
Source: Polymers (Basel). 2022 Nov 27;14(23):5162. doi: 10.3390/polym14235162 (PMC9739291; doi:10.3390/polym14235162)
Supplement: Supplementary file 1 [file polymers-14-05162-s001.zip › polymers-2035842-supplementary.pdf]

Supplementary Materials

Fabrication of Superhydrophobic/Superoleophilic Bamboo Cellulose Foam for Oil/Water Separation

Chun-Hua Liu †, Jiao-Ping Shang †, Xing Su, Shuang Zhao, Yun Peng * and Yi-Bao Li *

Engineering Research Center of Jiangxi Province for Bamboo-based Advanced Materials and Biomass Conversion, College of Chemistry and Chemical Engineering, Gannan Normal University, Ganzhou 341000, China

* Correspondence: pengyun@buaa.edu.cn (Y.P.); liyb@gnnu.cn (Y.-B.L.)

† These authors contributed equally to this work.

Table S1. Bleached bamboo pulp ingredients

Mη (w)	α-cellulose (%)	Hemicellulose (%)	Ash (%)	Fe^{3+} (mg/kg)	Whiteness (%)	Dichloromethane extract (%)
19	86	16.6	1.0	58.	86.6	6.0

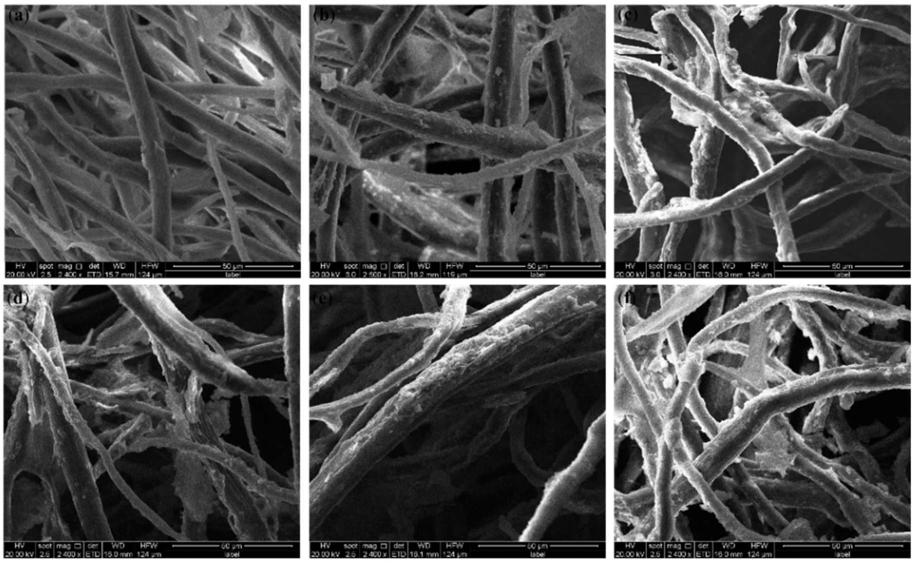

Figure S1. SEM images of original foam (a) and modified foams with various concentration of CHDTMS including : 1 mmol/L (b), 4 mmol/L (c), 7 mmol/L (d), 10 mmol/L (e), and 13 mmol/L (f)

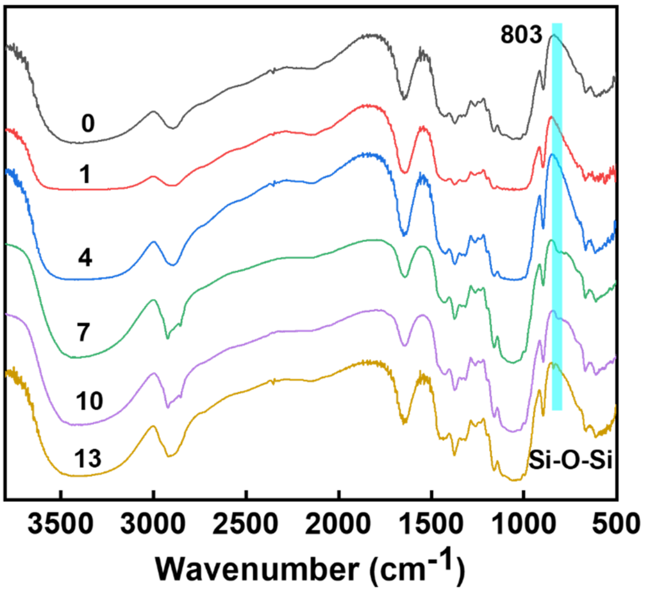

Figure S2. FTIR spectra of original foam and modified foams with various concentration of CHDTMS

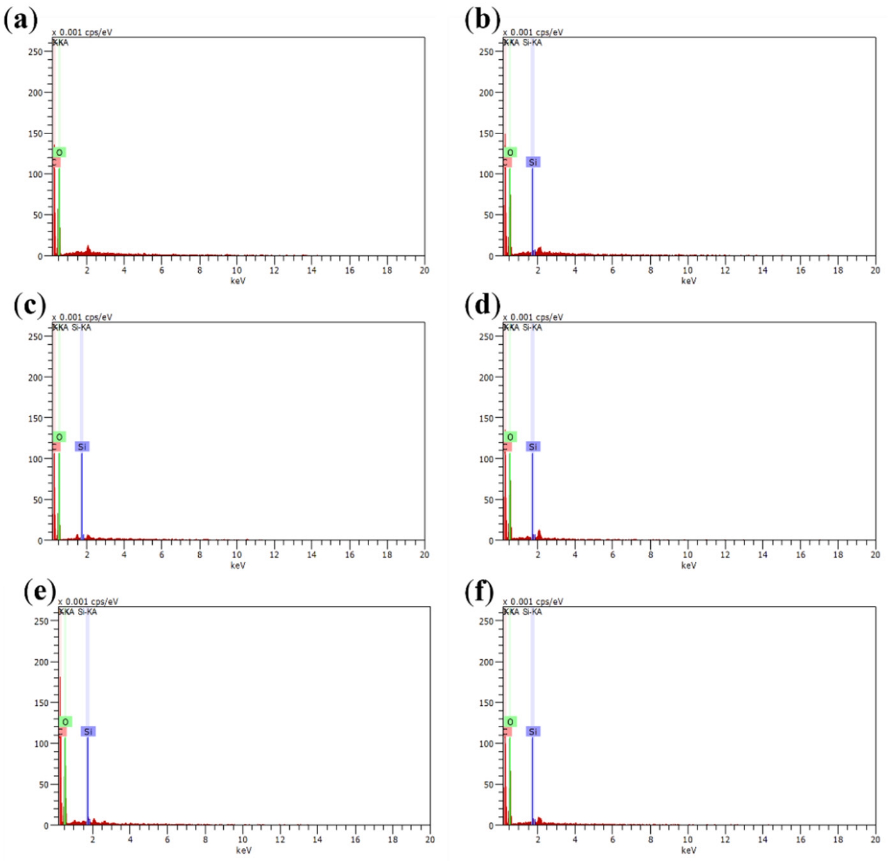

Figure S3. EDS data of original foam (a) and modified foams with various concentration of C_{HDTMS}: (b) 1 mmol/L, (c) 4 mmol/L, (d)7 mmol/L, (e) 10 mmol/L, and (f) 13 mmol/L

Table S2. EDS data of the Si content in of raw foam and modified foams with various concentration of C_{HDTMS}

**Element**	Line Type	0 mmol/L	1 mmol/L	4 mmol/L	7 mmol/L	10 mmol/L	13 mmol/L
**C (wt%)**	K-series	58.14	33.6	38.44	46.69	46.1	41.74
**O (wt%)**	K-series	72.76	40.94	45.65	53.12	54.86	41.74
**Si (wt%)**	K-series	0	0.21	0.23	0.3	0.35	0.47

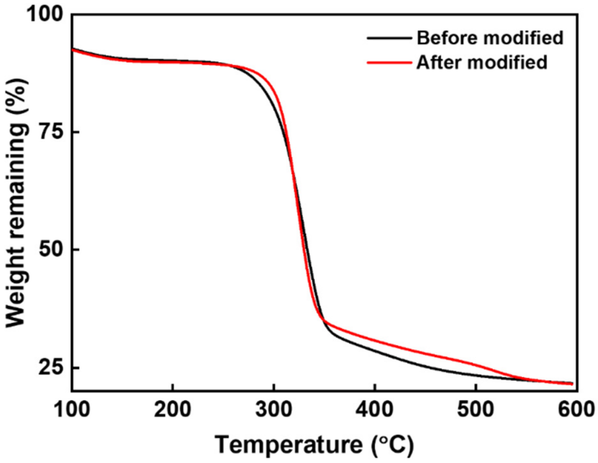

Figure S4. TG of bamboo cellulose foams before (black line) and after (red line) modification.

Table S3. The changes of after modification foams with different C_{HDTMS} in weight, contact angle and oil absorption

$C_{(HDTMS)}$ mmol/L	before modification (g)	after modification (g)	$\Delta m_{(After - before)}$ (g)	adsorption rate (g/g)	Contact Angle (º)
1	0.2135	0.22367	0.01017	11	160.72
4	0.3008	0.31228	0.01148	12.98	163.44
7	0.2407	0.25401	0.01331	11.38	164.39
10	0.2479	0.26452	0.01662	10.75	164.71
13	0.2121	0.23604	0.02394	11.31	165.02

Video S1

The observation of heavy oil adsorption of the super-wetting bamboo cellulose foam through a camera.

Video S2

The observation of light oil adsorption of the super-wetting bamboo cellulose foam through a camera.
